# Utilizing C-Reactive Protein (CRP) and CRP Ratios for Early Detection of Postoperative Complications Following Rectal Cancer Surgery

**DOI:** 10.3390/life14111465

**Published:** 2024-11-12

**Authors:** Valentin Calu, Catalin Piriianu, Adrian Miron, Valentin Titus Grigorean

**Affiliations:** 1 Elias University Emergency Hospital, 011461 Bucharest, Romania; valentin.calu@umfcd.ro (V.C.); adrian.miron@umfcd.ro (A.M.); 2 Department of Surgery, Carol Davila University of Medicine and Pharmacy, 020021 Bucharest, Romania; grigorean.valentin@yahoo.com; 3 “Bagdasar-Arseni” Clinical Emergency Hospital, 12 Berceni Road, 041915 Bucharest, Romania

**Keywords:** rectal cancer surgery, C-reactive protein (CRP), postoperative complications, surgical approach, CRP ratios, healthcare costs, estimated blood loss (EBL)

## Abstract

Background: Surgical treatment for rectal cancer may cause complications that exacerbate the condition, lengthen hospital stays, and raise healthcare costs. Rectal cancer surgery complications can be detected immediately with postoperative C-reactive protein (CRP) monitoring. Elevated levels of CRP indicate the presence of inflammation and can serve as a predictive factor for future outcomes. Methods: A retrospective cohort analysis was performed on 67 rectal cancer surgery patients. Prior to and after surgery, CRP levels were measured on days 1–3 and 4–7. Postoperative complications were linked to CRP, surgical approach (open, laparoscopic, conversion), and patient characteristics. This study included *t*-tests, chi-square tests, Mann–Whitney U tests, ANOVA, Pearson and Spearman correlation analyses, and logistic regression. Results: There was a significant correlation between high levels of CRP on postoperative days 4–7 and the development of problems (*p* < 0.001). The ratios of CRP/albumin and CRP/platelet were highly predictive of problems over this period (*p* = 0.000033). Patients who encountered problems had a notably greater estimated blood loss (*p* = 0.0086). Logistic regression analysis demonstrated a statistically significant relationship between higher Charlson Comorbidity Index (CCI) scores and an elevated probability of experiencing problems (*p* = 0.0078). Moreover, patients who underwent laparoscopic surgery but had to be converted to an open method saw a notably greater incidence of complications (*p* = 0.0022). From a financial standpoint, the average cost per patient with complications was EUR 1128.75, resulting in a total cost of EUR 44,021.25 for all 39 patients. Conversely, patients who did not experience any difficulties had a cost of EUR 731.25 per patient, resulting in a total of EUR 20,475.00 for all 28 patients. Conclusions: Regularly monitoring CRP, particularly between days 4 and 7 following surgery for rectal cancer, can promptly identify any complications. Monitoring CRP levels and promptly managing any abnormalities can enhance surgical outcomes and reduce healthcare costs.

## 1. Introduction

Rectal cancer, a specific kind of colorectal cancer (CRC), is a major global health issue that impacts almost 1 million people each year. It has unique epidemiological and clinical features when compared to colon cancer [1]. There has been a rise in the occurrence of rectal cancer among people who are under the age of 50, even though the general death rates in Western countries have been decreasing because of successful screening programs [2].

Rectal cancer surgery is linked to a range of postoperative complications that can greatly affect patient outcomes and quality of life. Roughly half of patients encounter problems, which not only raise healthcare expenses but also impact long-term cancer treatment results by potentially postponing or stopping essential chemotherapy [3]. Complications in this context are influenced by various factors, including patient-specific characteristics such as age, gender, tumor location, and body mass index (BMI), as well as surgical procedures and perioperative care practices such as fluid treatment and mechanical colon cleaning [4].

Postoperative complications after rectal cancer surgery can be detected early using CRP and its ratios. Biomarkers can predict infection complications, prognosis, and hospitalization costs. Adding clinical criteria like the CCI to CRP readings can improve postoperative prediction. CRP may predict sepsis outcomes [5].

Elevated levels of CRP, specifically the fluctuation in CRP levels throughout the days following surgery, have been demonstrated to be indicative of significant problems. An independent risk factor for significant complications was found by identifying a proportionate increase in CRP between postoperative days 1 and 4. This risk factor showed strong predictive potential, as indicated by a high area under the curve (AUC) value [6]. Integrating CRP with other indicators, such as white blood cell counts, improves the precision of predicting infection complications. This combination has demonstrated a notable level of sensitivity and specificity in forecasting problems, rendering it a helpful instrument in clinical environments [7].

The CRP/Albumin Ratio (CAR) after rectal cancer surgery has been found to be a reliable indicator of early problems [8]. The ratio mentioned, in addition to the preoperative fibrinogen/albumin ratio (FAR), offers a thorough assessment of the patient’s inflammatory and nutritional condition, which is crucial for predicting postoperative results. Long-term Prognosis: Increased postoperative CRP levels are linked to worse long-term results, such as worse overall survival and cancer-specific survival rates. This correlation emphasizes the significance of CRP as a predictive indicator beyond the initial time following surgery [9,10].

To summarize, CRP is a valuable and dynamic biomarker for predicting and treating postoperative problems in rectal cancer surgery. It aids in the prompt identification of problems, improves patient results, and has the potential to decrease hospital stays by enabling quick interventions.

This research assesses the predictive significance of C-reactive protein (CRP) levels and CRP ratios in the early identification of postoperative complications after rectal cancer surgery. This study analyzes a cohort of 67 patients who underwent rectal cancer surgery from 2020 to 2023, aiming to equip clinicians with effective tools for enhancing postoperative care via early intervention.

## 2. Materials and Methods

This study is a retrospective cohort study that included patients who underwent surgery for rectal cancer (Supplementary Materials Table S1). The study period encompassed the COVID-19 pandemic, during which the selection of surgical methods, namely laparoscopic surgery, was affected by the limitations and difficulties presented by the pandemic.

The inclusion criteria were individuals who were 18 years of age or older, had a confirmed diagnosis of rectal adenocarcinoma through histology, and had tumors placed within a range of 0 to 15 cm from the anal margin. The exclusion criteria included colon cancer with tumors placed more than 15 cm from the anal margin, insufficient medical records, and non-cancerous abnormalities of the rectum. 

The patients participated in the data collecting process after meeting the inclusion criteria and giving informed consent, as required by Law 95/2006, Law 46/2003 on patient rights, WHO No. 141:0/2016, and the Ministry of Health’s Order No. 482/2007, which was included in the admission files.

Amidst the COVID-19 pandemic, specifically in 2020 and early 2021, the choice to conduct laparoscopic surgery was approached carefully due to worries about the creation of aerosols and the possible transmission of the virus within operating rooms. Occasionally, these circumstances resulted in an increased rate of transition to open surgery. The selection of the surgical approach was determined by factors such as tumor attributes, the proficiency of the surgeon, and the preference of the patient.

The patients had one of three surgical procedures: Anterior Resection (AR), Abdominoperineal Excision of the Rectum (APER), or Hartmann Procedure. All procedures were conducted with the aim of curing the patient and according to established oncological guidelines, including the use of total mesorectal excision (TME) when necessary.

Preoperative and postoperative CRP levels were examined on specific days: day 1, day 3, and day 5. For patients who were in the hospital longer, further measures were taken on day 7. CRP levels were measured using a high-sensitivity immunoassay. The CRP/Platelet ratio (CPR) was computed for each day following the surgery to serve as an indicator of inflammation. Additionally, the CRP/Lymphocyte ratio (CLR) and CRP/Neutrophil ratio (CNR) were computed to investigate their correlation with surgical complications.

CRP levels were assessed utilizing the CRP Vario assay, a latex immunoturbidimetric test conducted on the ARCHITECT C Systems (Abbott Laboratories). This assay employs two reagents: glycine buffer (pH 7.0) and anti-CRP polyclonal antibodies adsorbed onto latex particles, facilitating accurate quantification of CRP in serum or plasma. Blood samples were obtained through standard venipuncture methods and processed by permitting clot formation, subsequently followed by centrifugation. The high-sensitivity mode was utilized, enabling precise detection over an extensive range of CRP concentrations. Samples were maintained at 2–8 °C for a maximum of 15 days, or at −20 °C for extended storage of up to one year. Reagents exhibited stability for a duration of 60 days while onboard the analyzer, with recalibration conducted if there were indications of compromised reagent integrity. This methodology facilitated a reliable evaluation of CRP dynamics concerning postoperative complications.

Demographic information of patients, such as age, gender, body mass index (BMI), and comorbidities, was obtained from electronic medical records. Information regarding tumor attributes, including dimensions, level of differentiation, and proximity to the anal verge, was also documented. The specifics of the operation, such as the projected amount of blood lost, the duration of the surgery, and whether or not a blood transfusion was required during the procedure, were recorded. The Clavien–Dindo classification system was used to categorize complications following surgery. 

Statistical analyses were conducted utilizing SPSS version 28.0 (IBM, Armonk, NY, USA) and Origin Pro 2018 software. Logistic regression models predicted postoperative complications using CRP levels and CRP ratios. Chi-square tests, *t*-tests, Mann–Whitney U tests, and ANOVA were employed as appropriate to evaluate group differences. A *p*-value below 0.05 was deemed statistically significant. 

The main focus was on the incidence of postoperative complications, such as surgical site infections, anastomotic leaks, and other significant issues. Additional outcomes examined were the duration of hospitalization and the financial burden related to complications after surgery. Costs were determined by multiplying the number of days spent in the hospital by the average daily cost of EUR 75.

Descriptive statistics were used to concisely describe the patient characteristics, details of the procedure, and results after the operation. The display of continuous data included measures of central tendency such as means with standard deviations or medians with interquartile ranges, depending on their distribution. The categorical variables were summarized by calculating the frequency and percentage distributions. The comparison analyses utilized *t*-tests or Mann–Whitney U tests for continuous variables and chi-square or Fisher’s exact tests for categorical variables. An analysis of variance (ANOVA) was used to examine the levels of CRP among different surgical procedures. A logistic regression analysis was performed to identify the independent variables that can predict postoperative complications. 

The study incorporated the following covariates: levels of CRP, the CRP ratios, the surgical method employed, and patient demographics. The measure of the strength of connections was determined by computing odds ratios (ORs) along with 95% confidence intervals (CIs). A *p*-value below 0.05 was considered statistically significant.

Postoperative complications were categorized according to the Clavien–Dindo system, a standardized and validated approach commonly employed in surgical research to assess the severity of complications [11].

## 3. Results

### 3.1. Preoperative Factors

#### 3.1.1. Patient Age

The study involved a cohort of 67 individuals who underwent surgical treatment for rectal cancer between 2020 and 2023. The average age of the patients was 64.78 years, with a range of 39 to 84 years. Out of the entire group, 39 patients, accounting for 58.2% of the total, encountered postoperative obstacles, whereas 28 patients, making up 41.8% of the total, did not experience any complications. The demographic distribution showed that older patients had a higher occurrence of complications, with an average age of 67.33 years in the group with complications compared to 61.21 years in the group without issues. The *t*-test analysis of patient ages, distinguishing between individuals with complications and those without, yielded a *t*-statistic of 2.32 and a *p*-value of 0.024. The average age difference between the two groups is statistically significant, indicating that patients with complications are generally older.

#### 3.1.2. Patient Gender

In the study cohort of 39 male patients (58.2%) and 28 female patients (41.8%), the likelihood of encountering postoperative problems was 69.2% for men and 42.9% for females. In particular, 27 male patients experienced issues, whereas only 12 female patients faced issues. The risk ratio (RR) for males relative to females was determined to be 1.62, signifying that male patients were 1.6 times more predisposed to postoperative problems than female patients. This indicates that male patients may possess an elevated likelihood and risk of experiencing complications following surgery.

#### 3.1.3. Surgery Type

Of the 43 patients who underwent AR, 23 (53.5%) had complications. Among the 18 patients who had APER, 12 (66.7%) experienced complications. An exceptionally high incidence of complications was noted in patients undergoing Hartmann procedures and APER, with more than two-thirds of individuals in these cohorts experiencing issues. All conversions experienced complications.

#### 3.1.4. Tumor Location (Distance from the Anus)

The logistic regression analysis revealed a negative correlation between the distance from the anus and the likelihood of encountering specific postoperative complications. Remarkably, there is a notable reduction in the probability of experiencing bleeding (coefficient: −0.78) and intestinal obstruction (coefficient: −0.56) as the distance of the tumor increases. The average distance for patients experiencing issues was 2.83 cm with a standard deviation of 3.11 cm. In contrast, for patients without complications, the average distance was 4.26 cm with a standard deviation of 4.30 cm. These data suggest that the proximity of the tumor to the anus is a crucial factor in predicting certain complications following surgery.

#### 3.1.5. Preoperative Charlson Comorbidity Index (CCI) Scores and Comorbidities 

**Figure 1:**
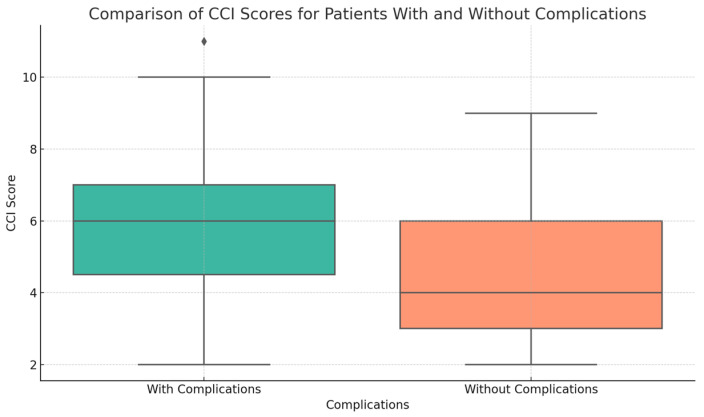
Correlation of Charlson Comorbidity Index (CCI) Scores with Postoperative Complications—This graphic illustrates the correlation between elevated CCI scores and postoperative complications. Patients experiencing complications exhibited higher mean CCI scores (5.92 ± 2.31) in contrast to those without complications (4.43 ± 1.87), underscoring the influence of comorbidity on surgical outcomes. Logistic analysis indicates that a one-unit increase in CCI results in a 0.35 increase in the log odds of complications (*p* = 0.010).

The investigation we conducted showed clear connections between the CCI and the occurrence of postoperative complications (Figure 1). The Mann–Whitney U test revealed a statistically significant difference in the CCI scores between patients with complications (mean: 5.92, SD: 2.31) and those without issues (mean: 4.43, SD: 1.87), with a *p*-value of 0.0078.

Logistic regression analysis revealed a statistically significant relationship between the rise in the CCI score and the increase in the log odds of suffering complications. Specifically, each unit increase in the CCI score corresponded to a 0.35 increase in the log odds of complications (*p* = 0.010). The 95% confidence interval for this increase in log odds is between 0.082 and 0.610, which further supports the robust association between higher CCI scores and an increased likelihood of postoperative complications.

The results emphasize the significance of including the CCI in preoperative evaluations, since a greater load of comorbidities, indicated by higher CCI scores, substantially raises the probability of postoperative problems. This emphasizes the necessity of thorough assessment and control of concurrent medical conditions to enhance the results of surgical procedures.

An examination of CCI scores across various age groups demonstrates a distinct pattern of escalating comorbidity burden with advancing age. Individuals younger than 50 years old had the lowest average CCI score of 2.00, with very little deviation. Conversely, those between the ages of 50 and 64 exhibited a higher average CCI score of 4.62, indicating a moderate rise in the presence of several medical conditions. The CCI score showed a steady increase in the age range of 65–74, with an average of 5.70, and reached its highest point in individuals aged 75 and beyond, who had an average score of 7.06. This pattern emphasizes the increasing weight of several health conditions as patients get older, emphasizing the significance of taking age-related health conditions into account during preoperative evaluations and planning. 

The incidence of postoperative complications exhibited a significant correlation with the prevalence of comorbidities among patients. Individuals experiencing complications had an average of 3.08 comorbidities (standard deviation: 1.66), whereas those without complications had an average of 2.18 comorbidities (standard deviation: 2.00). The median number of comorbidities in the complication group was 3, whereas in the non-complication group, it was 2.

The Mann–Whitney U test indicated a statistically significant difference (*p* = 0.0218) in the number of comorbidities between the two groups. Logistic regression analysis indicated a significant correlation between the number of comorbidities and an increased likelihood of complications. This underscores the essential requirement for thorough preoperative evaluations to identify and manage various health concerns, thereby reducing the risk of postoperative complications. The findings indicate that patients with three or more comorbidities may require enhanced monitoring to reduce the risk of negative postoperative outcomes. 

#### 3.1.6. Smoking

The data analysis reveals a significant association among smoking, intraoperative blood loss, and postoperative complications. Among the patients assessed, a higher percentage of smokers (16 out of 22, or 72.7%) experienced issues compared to non-smokers (23 out of 45, or 51.1%). Smokers exhibited a statistically significant greater estimated blood loss (mean EBL: 252.32 mL, SD: 124.99 mL) in comparison to non-smokers (mean EBL: 212.22 mL, SD: 148.15 mL). Furthermore, patients experiencing significant blood loss were more susceptible to complications. This suggests a credible cause-and-effect relationship wherein smoking results in increased bleeding during surgery, thereby elevating the risk of postoperative complications. The statistical evidence underscores the significant role of smoking as a modifiable risk factor, highlighting the potential benefits of smoking cessation programs in reducing intraoperative blood loss and the likelihood of complications, hence improving overall patient outcomes.

#### 3.1.7. Tumor Marker Levels (CEA and CA 19-9)

The levels of carcinoembryonic antigen (CEA) and carbohydrate antigen 19-9 (CA 19-9) markers differed between patients who experienced postoperative complications and those who did not. The mean CEA level for individuals experiencing complications was 19.70 ng/mL (SD: 70.13), with a median value of 3.19. Conversely, individuals without complications exhibited a higher mean CEA level of 348.52 ng/mL (SD: 1823.90) and a median of 2.52. Although certain individuals without complications exhibited markedly elevated CEA levels, the majority displayed values similar to those with complications, indicating the existence of outliers within the non-complication cohort. The average CA 19-9 level for patients experiencing complications was 57.81 U/mL (SD: 185.54), with a median value of 10.30 U/mL. The non-complication group exhibited a mean of 235.95 U/mL (SD: 1156.82) and a median of 12.66. The results indicate that elevated levels of CEA and CA 19-9 may correlate with complications; however, the presence of outliers in the non-complication group complicates the ability to reach definitive conclusions.

#### 3.1.8. American Society of Anesthesiologists (ASA) Score

The preoperative health status, indicated by the ASA score, significantly affects the likelihood and severity of postoperative complications, as classified by the Clavien–Dindo system. Patients with lower ASA scores, indicative of fewer comorbidities or less severe systemic disease, exhibited a greater likelihood of encountering no complications (Clavien–Dindo Grade 0). Among patients with Clavien–Dindo Grade 2 complications, 23 had an ASA score of 3, while 6 had an ASA score of 4, indicating a higher prevalence of preoperative health concerns. The severity of sequelae markedly increased in patients classified as Clavien–Dindo Grades 3 and 4a. Patients presented with ASA scores of 3 or 4, signifying an increased probability of life-threatening conditions and the necessity for critical care.

This analysis demonstrates the correlation between elevated preoperative ASA scores, indicative of more severe systemic disease, and an increased risk of significant postoperative complications, as classified by the Clavien–Dindo system. This highlights the importance of thorough preoperative assessment and management to mitigate the risks linked to high ASA scores.

#### 3.1.9. Preoperative CRP Levels and Ratios

**Figure 2:**
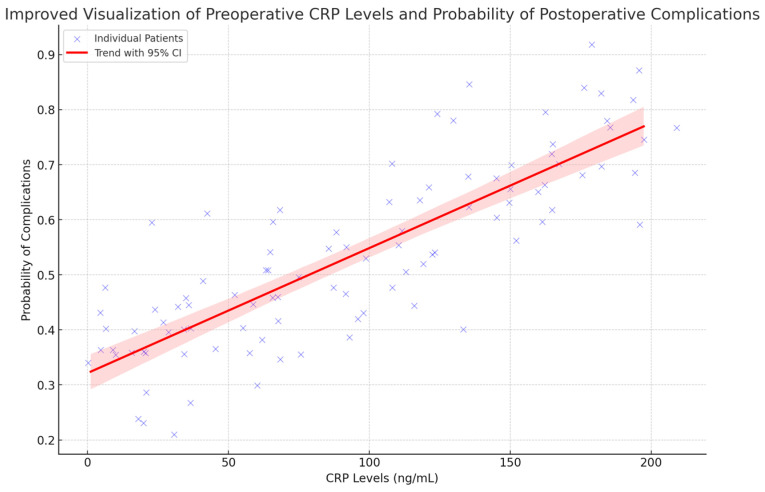
Preoperative CRP Levels as Predictors of Postoperative Complications—This scatter plot illustrates the significant correlation between elevated preoperative CRP levels and an increased likelihood of postoperative complications. The blue points represent individual patients, with jitter included to demonstrate data variability. The trend line and 95% confidence interval demonstrate a significant correlation between CRP levels and the risk of complications, underscoring the utility of CRP as a predictive tool. *p* < 0.05.

The logistic regression analysis demonstrated a statistically significant relationship between preoperative CRP levels and the probability of experiencing surgical complications. This figure (Figure 2) demonstrates the correlation between preoperative CRP levels and the likelihood of postoperative complications. The blue points denote individual patients, with jitter incorporated to illustrate variability. The red line illustrates the relationship between CRP levels and the probability of complications, accompanied by a shaded area representing the 95% confidence interval. Elevated CRP levels are typically linked to a higher likelihood of complications. This highlights the potential usefulness of CRP in assessing the risk of complications before surgical interventions.

Preoperatively, the ratios of CLR, CAR, CPR, CRP/monocyte ratio (CMR), and CNR were examined to forecast postoperative complications. The descriptive data revealed certain disparities between patients with and without complications; however, none of them reached statistical significance. The average CLR was 35.91 in individuals with difficulties, compared to 25.10 in those without. However, the *t*-test did not reveal a statistically significant difference (*p* = 0.585). The ratios of CAR, CPR, CMR, and CNR showed no significant differences across the groups, with *p*-values far above 0.05. All logistic regression models had non-significant coefficients and *p*-values, therefore showing that none of these CRP ratios were predictive of any difficulties. The data suggest that CRP ratios may indicate the presence of inflammatory conditions, but they cannot be relied upon to accurately predict postoperative problems in this particular group.

### 3.2. Factors Analyzed During Surgery

#### 3.2.1. Estimated Blood Loss (EBL)

**Figure 3:**
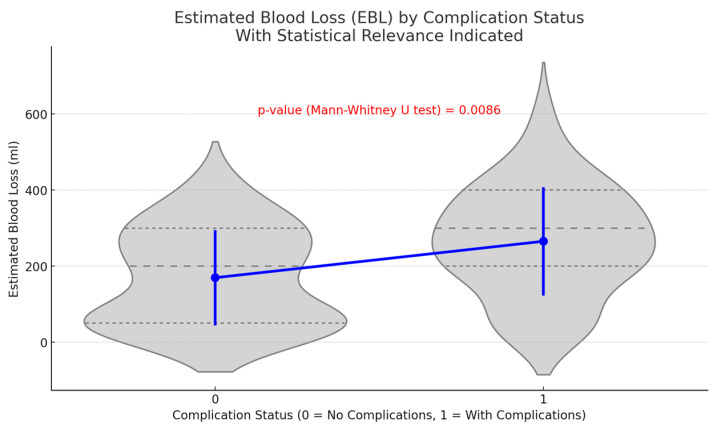
The influence of Estimated Blood Loss (EBL) on postoperative complications—This figure illustrates the comparison of estimated blood loss (EBL) between patients with complications and those without. The data indicate that patients with complications experienced a significantly higher mean EBL of 265.41 ± 140.62 mL in contrast to a mean EBL of 169.64 ± 124.23 mL for patients without complications. The difference was statistically significant (*p* = 0.0046), suggesting that increased intraoperative blood loss may elevate postoperative risk.

A notable disparity in EBL during surgery was identified between individuals experiencing postoperative complications and those who did not. Individuals facing challenges demonstrated a significantly elevated average EBL of 265.41 mL (standard deviation: 140.62 mL), in contrast to those without complications, who recorded an average EBL of 169.64 mL (standard deviation: 124.23 mL). Statistical analyses, comprising a *t*-test (*p* = 0.0046) and a Mann–Whitney U test (*p* = 0.0086), demonstrate that this disparity is statistically significant (Figure 3). The results indicate a correlation between increased blood loss during surgery and a heightened risk of postoperative complications.

#### 3.2.2. Operative Time

The influence of operative time on postoperative outcomes was analyzed. The average operative time was 313.15 min for patients with complications and 311.96 min for those without, showing no significant difference (*p* = 0.958). Logistic regression analysis indicated no significant correlation between operative time and the probability of complications (*p* = 0.960). The findings indicate that operative time is not a significant factor influencing postoperative complications in this cohort, underscoring the necessity for a comprehensive evaluation of risk factors.

#### 3.2.3. Surgical Approach

A chi-square test of independence was performed to assess the relationship between the surgical approach type (open, laparoscopic, or conversion) and the incidence of postoperative complications. The findings indicated a statistically significant relationship between the surgical approach and complications, χ^2^ (2, *N* = 67) = 12.24, *p* = 0.0022. This indicates that the probability of complications differs markedly depending on the surgical technique employed.

**Figure 4:**
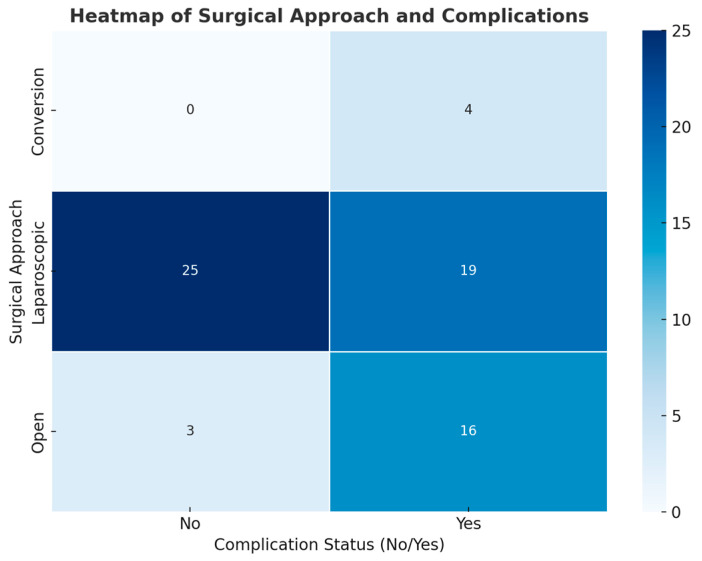
Postoperative Complications Distribution by Surgical Approach: Laparoscopic, Open, and Conversion—The heatmap illustrates the incidence of complications according to the surgical technique employed (laparoscopic, open, or conversion). Patients who underwent laparoscopic surgery experienced a lower complication rate of 43% in contrast to 84% for open surgery and 100% for conversion cases. Chi-square analysis reveals a significant association between surgical method and complication rates (*p* = 0.0022), indicating the advantages of laparoscopic techniques when applicable.

Patients who underwent laparoscopic surgery exhibited a lower observed complication rate of 43% in contrast to an 84% rate for those undergoing open surgery and a 100% rate for conversions from laparoscopic to open surgery (Figure 4). The anticipated incidence of complications among patients varied significantly from the actual observed figures, especially within the open and conversion groups, where the occurrence of complications exceeded expectations. The findings suggest that laparoscopic surgery is linked to a lower incidence of postoperative complications in comparison to open and conversion procedures.

### 3.3. Postoperative Factors

#### 3.3.1. Postoperative Complications

Data indicate that 58.2% of the 67 individuals who underwent rectal surgery experienced complications post-operation. Surgical site infections (SSI) were the most prevalent type of infection, occurring in 22 cases. Furthermore, four occurrences of both bleeding and urinary tract infections (UTI) were noted. Notable complications included delayed fistula formation in three cases, pneumonia in three cases, dehydration in two cases, and intestinal obstruction in two cases. Additionally, several rare issues were observed, with each affecting only a single patient. The conditions included COVID-19, Clostridium infection, Takotsubo cardiomyopathy, STEMI, and acute urine retention (AUR). The distribution of complications indicates that most cases were of moderate severity (Grade 2), while a significant proportion were classified as severe (Grade 3a and 4a), substantially impacting the patient’s postoperative recovery.

#### 3.3.2. Postoperative CRP Levels and Ratios

No significant difference in CRP levels was observed between patients who experienced complications and those who did not during the postoperative period, specifically on days 1–3. The mean CRP level was 95.36 mg/L (standard deviation: 50.78) in the group with complications and 88.59 mg/L (standard deviation: 68.45) in the group without complications (*p* = 0.269), indicating that CRP levels lack predictive value for complications during this early period. 

**Figure 5:**
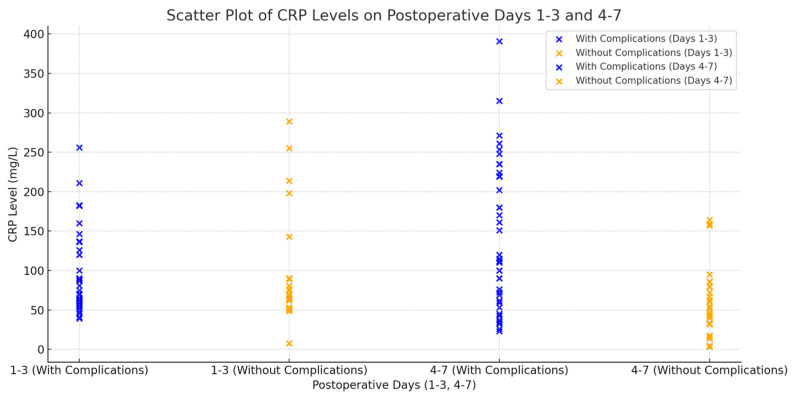
Comparative Analysis of Postoperative CRP Levels in Patients with and Without Complications During Days 1–3 and 4–7 Following Rectal Cancer Surgery—This box plot illustrates the comparison of CRP levels on postoperative days 1–3 and 4–7 between patients experiencing complications and those without complications. Initial CRP levels (Days 1–3) were comparable; however, patients with complications exhibited significantly higher CRP levels on Days 4–7, indicating this later timeframe as crucial for monitoring (*p* = 0.000045).

Individuals experiencing complications on days 4–7 post-surgery exhibited markedly elevated CRP levels (140.93 mg/L, SD: 91.80) in contrast to those without complications (58.44 mg/L, SD: 44.31). The observed difference was statistically significant, with a *p*-value of 0.000045 (see Figure 5). The findings suggest that monitoring CRP levels within 4–7 days post-surgery is a more effective approach for predicting postoperative complications.

Similarly, patients experiencing difficulties exhibited a significantly elevated CAR ratio between postoperative days 4–7, averaging 49.92 (SD: 34.21), in contrast to 18.17 (SD: 14.48) observed in patients without complications. The difference was statistically significant, as indicated by a *t*-test (*p* = 3.32 × 10^−5^), with a mean difference of 31.75 (95% CI: 19.45–44.05). Logistic regression analysis indicated that the CAR ratio is a significant predictor of complications (*p* = 0.000033), with an odds ratio of 2.15 (95% CI: 1.47–3.14) for each unit increase in the CAR. 

The CPR exhibited notable differences and demonstrated predictive efficacy. Patients experiencing complications within the initial three days post-surgery exhibited a significantly elevated CPR (mean: 0.536, SD: 0.403) relative to those without complications (mean: 0.352, SD: 0.200). This was confirmed by a *t*-test (*p* = 0.0466), revealing a mean difference of 0.184 (95% CI: 0.002–0.366). The CPR emerged as a significant predictor of complications during this period, exhibiting an odds ratio of 1.86 (95% CI: 1.02–3.38) for each unit increase in the CPR. 

**Figure 6:**
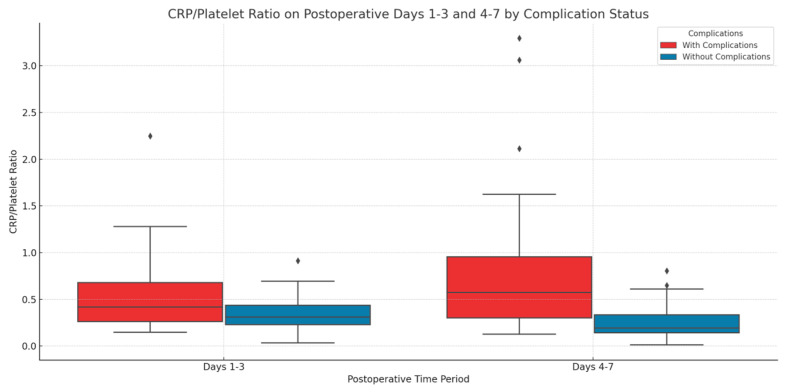
The predictive value of the CRP/platelet ratio (CPR) on days 1–3 and 4–7 for postoperative complications following rectal cancer surgery—This box plot illustrates the CRP/Platelet Ratio (CPR) during postoperative days 1–3 and days 4–7. Increased CPR on Days 4–7 was significantly correlated with complications, indicating CPR’s function as a marker of inflammation and postoperative risk (*p* = 1.59 × 10⁻⁵). Logistic regression analysis indicated that CPR is a significant predictor of complications during this timeframe.

The difference in CPR between patients with complications and those without became more pronounced during days 4 to 7 post-surgery. The mean CPR for patients with complications was 0.801 (SD: 0.727), while for patients without complications, the mean ratio was 0.252 (SD: 0.200). The observed difference was statistically significant, indicated by a *t*-test (*p* = 1.59 × 10^−5^), with a mean difference of 0.549 (95% CI: 0.321–0.777). Logistic regression analysis revealed that CPR during days 4–7 significantly predicted complications, yielding an odds ratio of 2.73 (95% CI: 1.89–3.95) for each unit increase (Figure 6).

**Figure 7:**
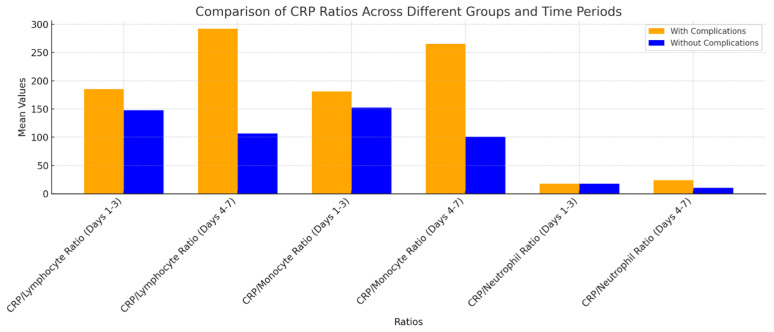
Analysis of CRP Ratios (CLR, CMR, CNR) in Patients With and Without Complications During Postoperative Days 1–3 and 4–7—This bar chart presents a comparison of the CRP/Lymphocyte Ratio (CLR), CRP/Monocyte Ratio (CMR), and CRP/Neutrophil Ratio (CNR) among patients with and without complications during postoperative days 1–3 and 4–7. Patients experiencing complications demonstrate elevated ratios, especially between days 4 and 7, with the CRP/Monocyte and CRP/Lymphocyte ratios reflecting the most significant increases. The findings indicate that increased CRP ratios during this critical period may be useful indicators for identifying patients at elevated risk for complications.

Patients who experienced complications showed significant increases in the CLR and CMR from postoperative days 4 to 7. Both ratios were identified as significant indicators of challenges. The findings demonstrate that CRP and its associated ratios serve as significant predictors for anticipating post-operative complications, especially during the critical period of days 4–7 post-surgery. The CNR exhibited a significant increase in individuals experiencing complications between days 4 and 7 (*p* = 4.30 × 10^−5^), thereby further confirming its prognostic potential (Figure 7). This tendency suggests that these ratios may serve as valuable indicators for postoperative complications, especially in the later stages of the recovery process.

### 3.4. Effects of Postoperative Complications

#### 3.4.1. Duration of Hospital Stay

An analysis of hospital stay durations reveals significant differences between patients with postoperative complications and those without, highlighting implications for medical care and financial considerations. The patients experiencing complications had an average hospitalization duration of 15.05 days, accompanied by a standard deviation of 8.15. The median duration was 13 days, while the maximum duration reached 55 days. Conversely, patients without complications had a notably reduced average hospitalization duration of 9.75 days (standard deviation: 2.44). The median duration was 9 days, with the maximum stay recorded at 17 days. The findings indicate that surgical complications are associated with prolonged and unpredictable hospitalizations, which directly impact patient recovery times and the overall resource utilization in healthcare facilities.

#### 3.4.2. Financial Costs

**Figure 8:**
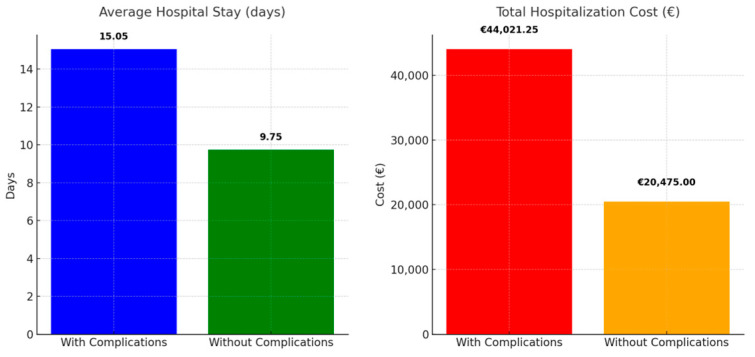
Analysis of Average Length of Hospital Stay and Total Hospitalization Expenses in Patients with Complications Versus Those Without—The bar chart on the left illustrates the average duration of hospital stays for patients with complications, which is 15.05 days, compared to 9.75 days for those without complications. Patients experiencing complications exhibited extended durations of hospitalization. The chart on the right illustrates total hospitalization costs, revealing that patients with complications incur significantly higher expenses (EUR 44,021.25) than those without complications (EUR 20,475.00). The findings underscore the significant resources and financial burden linked to postoperative complications.

Prolonged hospitalization due to complications significantly elevates costs from a financial perspective. The average daily cost is EUR 75, resulting in an estimated total cost of EUR 1128.75 per patient with complications in Romanian public hospitals. The cumulative cost for the 39 patients experiencing complications is EUR 44,021.25. Conversely, patients without any difficulties incur a charge of EUR 731.25 each, leading to an aggregate cost of EUR 20,475.00 for the 28 patients (Figure 8).

This disparity highlights the additional burden that complications impose on the healthcare system and underscores the importance of effective preoperative care and postoperative monitoring to reduce the incidence of complications, thereby mitigating both clinical and financial impacts on healthcare services. This underscores the essential need for techniques aimed at reducing complications to improve patient outcomes and lessen the financial strain on healthcare systems.

Patients experiencing postoperative complications demonstrated increased estimated blood loss, higher concentrations of CEA and CA 19-9, and prolonged hospital stays, with an average duration of 15.05 days (Factors and signs related to postoperative complications in clinical and surgical settings—Supplementary Materials Table S2). Patients were often categorized into higher Clavien–Dindo grades, signifying the presence of more severe outcomes. Data indicate that increased blood loss, elevated tumor markers, and prolonged hospital stays are significant predictors of more severe surgical outcomes.

#### 3.4.3. Mortality

Mortality was observed solely in patients experiencing surgical complications, with both deceased individuals classified as ASA 4. This grade signifies a significant systemic illness that presents a continuous risk to life. The existence of these complex preoperative conditions likely heightened the risk of complications and subsequent mortality. The findings underscore the substantial influence of preoperative health status, particularly elevated ASA scores, on predicting negative surgical outcomes, including mortality.

## 4. Discussion

Multiple studies have investigated the association between CRP levels, CRP ratios, the CCI, and hospitalization costs in identifying postoperative complications following rectal cancer surgery. CRP, a recognized biomarker for inflammation, is often elevated following surgery in instances of complications such as infections or anastomotic leaks [12]. Research demonstrates that CRP levels measured on postoperative days 3 to 5 can act as a predictive indicator for early detection, facilitating timely intervention [5,6]. The CCI serves as an important instrument, with elevated scores correlating to a greater risk of complications and extended hospitalizations, resulting in increased costs [7,9]. The integration of CRP and CCI facilitates a thorough risk assessment, thereby enhancing personalized postoperative care and optimizing resource allocation [13]. Nonetheless, challenges persist—CRP levels may vary due to non-surgical influences, and the CCI may not comprehensively encompass all pertinent patient attributes [8].

The expense associated with hospitalization is a major issue when it comes to the administration of rectal cancer surgery. Complications have a dual impact, negatively affecting both patient outcomes and significantly raising healthcare expenses as a result of prolonged hospital stays and the need for further treatments. By employing CRP and CRP ratios for early identification, healthcare systems have the potential to decrease these expenses by averting problems or lessening their intensity through prompt intervention [13,14]. Furthermore, the utilization of predictive models that include both CRP and CCI can enhance the allocation of resources and enhance cost-effectiveness in the provision of postoperative care [10]. These tools are valuable; however, they should be utilized in conjunction with clinical judgment and additional diagnostic methods. Subsequent investigations ought to concentrate on improving these models to increase their predictive precision and clinical applicability.

The use of CRP ratios, which assess CRP levels at different postoperative intervals, enhances the accuracy and precision of prognostic evaluations, providing a dynamic assessment of the patient’s inflammatory response [15,16]. 

The CAR levels post-surgery serve as a reliable indicator of early complications following rectal cancer surgery and can independently predict these complications [8,17,18,19,20]. The specified ratio, along with the preoperative FAR, provides a comprehensive view of the patient’s inflammatory and nutritional status, essential for anticipating complications. While CRP alone serves as a reliable indicator, incorporating additional markers like white blood cell counts can enhance predictive accuracy [21]. The combination of CRP and white blood cell count (WBC) on postoperative day 4 (POD 4) significantly improves the detection of complications, enhancing both sensitivity and specificity [7,22].

Increased concentrations of CRP correlate with heightened blood loss during surgical interventions and prolonged hospital stays, thereby directly impacting healthcare costs [23]. Monitoring CRP levels enables the early recognition and management of issues [5,13]. Although CRP is frequently the primary focus, numerous studies underscore the importance of comprehensive biomarker panels that include supplementary inflammatory markers and vital signs. This technique offers a comprehensive and holistic approach to postoperative care. Adopting a broader perspective enables more accurate predictions of future challenges and the formulation of tailored treatment strategies, potentially improving outcomes and reducing costs [14,15]. 

Our findings demonstrate that increased CRP levels and CRP-based ratios serve as significant predictors of postoperative complications in rectal cancer surgery. This is consistent with extensive research indicating that CRP serves as a reliable marker for the early detection of complications in colorectal procedures. Singh et al. [24] and Matthiessen et al. [25] established a strong correlation between elevated CRP levels and adverse outcomes, including anastomotic leaks and infectious complications, after colorectal surgery. This study enhances existing findings by concentrating on rectal cancer surgery, thereby affirming the predictive value of CRP within this specific surgical setting.

These findings indicate that routine monitoring of CRP after colorectal surgery may serve as an effective method for the early detection of complications, thereby enhancing patient outcomes.

## 5. Conclusions

Prompt recognition of postoperative complications following rectal cancer surgery is crucial for improving patient outcomes and reducing healthcare costs. Key indicators in this context are CRP levels, the CCI, and hospitalization costs. CRP functions as a valuable biomarker for predicting postoperative complications, while CCI assists in assessing the impact of comorbidities on patient outcomes and costs. This response analyzes the roles of CRP and CCI in early the identification of complications and their influence on hospitalization costs. 

CRP serves as a reliable marker for detecting infection issues post-colorectal surgery. Elevated CRP levels on specific postoperative days correlate with complications including pneumonia, wound infection, and leakage. We propose a CRP cut-off value of around 140 mg/L on postoperative days 4–7 as a threshold for worry. This figure is associated with a notable rise in the probability of problems among our sample. Nonetheless, additional confirmation in larger research is essential to determine a more definitive cut-off point.

This study introduces the CRP to platelet ratio (CPR) as a statistically significant predictor of complications within the first three days post-surgery (*p* = 0.0466). The CRP ratio serves as a more effective predictor, providing enhanced diagnostic accuracy relative to CRP alone. The CCI serves as a valid measure of healthcare costs, length of hospital stays, and mortality risk in colorectal cancer patients undergoing surgical procedures. Patients with a CCI score of 3 or higher incur greater costs and experience extended hospital stays.

While CRP and CCI are valuable for predicting postoperative complications and managing costs, it is essential to account for other factors, including patient demographics, surgical procedures, and institutional protocols, which may also influence outcomes. The prognostic significance of CRP varies according to the specific surgical procedure and patient characteristics, necessitating tailored strategies for different clinical contexts.

Future research should focus on enhancing these models to increase their practicality and cost-effectiveness, thereby improving patient care in rectal cancer surgery.

## Data Availability

Data supporting the reported results are available in the following datasets: Medline-PubMed: https://pubmed.ncbi.nlm.nih.gov/ (accessed on 1 June 2024), Cochrane Library: https://www.cochranelibrary.com/ (accessed on 1 June 2024), EMBASE: https://www.embase.com/ (accessed on 1 June 2024).

## Supplementary Materials

The following supporting information can be downloaded at: https://www.mdpi.com/article/10.3390/life14111465/s1, Table S1: Patient Demographics and Preoperative Characteristics; Table S2: Postoperative Outcomes and Complications.

## Abbreviations

CRP	C-reactive protein
CRC	Colorectal cancer
BMI	Body mass index
WHO	World Health Organization
APER	Abdominoperineal excision of the rectum
TME	Total mesorectal excision
AR	Anterior resection
STEMI	ST elevation myocardial infarction
SSI	Surgical site infections
UTI	Urinary tract infections
AUR	Acute urine retention
EBL	Estimated blood loss
ASA	American Society of Anesthesiologists
CCI	Charlson Comorbidity Index
HBP	High blood pressure
CAR	CRP/albumin ratio
FAR	Fibrinogen/albumin ratio
CPR	CRP/platelet ratio
CLR	CRP/lymphocyte ratio
CNR	CRP/neutrophil ratio
CMR	CRP/monocyte ratio
RR	Risk ratio
POD	Postoperative day
SD	Standard deviation
WBC	White blood cell
CEA	Carcinoembryonic antigen
CA 19-9	Carbohydrate antigen 19-9
