# Supplementary material for: Utilizing C-Reactive Protein (CRP) and CRP Ratios for Early Detection of Postoperative Complications Following Rectal Cancer Surgery
Source: Life (Basel). 2024 Nov 12;14(11):1465. doi: 10.3390/life14111465 (PMC11595844; doi:10.3390/life14111465)
Supplement: Supplementary file 1 [file life-14-01465-s001.zip › life-3200325-supplementary.pdf]

Table S1 - Patient Demographics and Preoperative Characteristics

Category	***Metric***	***With Complications***	***Without Complications***	***Results***
Age (years)	*Mean*	*67.33*	*61.21*	*Complications are more prevalent among older patients. The t-test analysis of patient ages, differentiating between individuals with complications and those without, produced a t-statistic of 2.32 and a p-value of 0.024. The average age difference between the two groups is statistically significant, suggesting that patients with complications tend to be older on average.*
	Standard Deviation	*10.34*	*11.09*	
	Median	*67*	*60*	
Gender	*Male*	*27*	*12*	*A greater percentage of male patients suffered complications.The chi-square test shows a **p-value of 0.056**, indicating a trend toward significance, but it does not quite reach the conventional threshold of 0.05.*
	Female	*12*	*16*	
BMI	*Mean*	*25.98*	*26.02*	*Both groups exhibited a mean and median BMI around 26. However, the group of patients with difficulties displayed a broader range and a bigger standard deviation.The t-test indicates no statistically significant difference in BMI between the two groups (p-value = 0.968).*
	Standard Deviation	*4.63*	*3.18*	
	Median	*26.12*	*25.69*	
Smoking status	*Smokers*	*16*	*6*	*The chi-square test indicates no statistically significant difference in smoking status between the two groups (p-value = 0.155), implying that smoking may not be strongly linked to postoperative complications in this dataset.*
	Non-smokers	*23*	*22*	
Comorbidities	*Mean*	*3.08*	*2.18*	*The t-test reveals a statistically significant difference in comorbidity counts between the two groups, with patients experiencing complications exhibiting a higher average number of comorbidities (p-value = 0.049).*
	Standard Deviation	*1.66*	*2.00*	
	Median	*3.0*	*2.0*	
Procedure	*Anterior resection*	*23*	*20*	*Anterior Resection was the predominant surgery in both groups, but its occurrence was higher among individuals with complications. The APER technique shown a greater frequency among individuals who experienced difficulties.*
	APER	*12*	*6*	
	Hartmann	*4*	*2*	
Surgical Approach	*Laparoscopic*	*19*	*25*	*The chi-square test reveals a statistically significant difference in surgical approach between the two groups (p = 0.002). This indicates that the surgical method may correlate with the incidence of postoperative complications.*
	Conversion	*4*	*0*	
	Open	*16*	*3*	
Operative Time (minutes)	*Mean*	*312.2*	*312.0*	*The t-test indicates no statistically significant difference in operative time between the two groups (p = 0.961), implying that operative time may not be a critical factor in predicting complications.*
	Standard Deviation	*110.0*	*75.1*	
	Median	*310.0*	*305.0*	
Distance from Anus (tumor) cm	*Mean*	*2.83*	*4.26*	*On average, tumors in patients who experienced complications were situated in closer proximity to the anus in comparison to those who did not encounter complications.*
	Standard Deviation	*3.11*	*4.30*	
	Median	*2.0*	*4.0*	

Preoperative Radiotherapy	*Yes*	*28*	*24*	*A greater proportion of patients who underwent preoperative radiation experienced complications in comparison to those who did not.*
	No	*11*	*4*	
American Society of Anesthesiologists (ASA)	*II*	*2*	*9*	*Most patients who encountered difficulties had an ASA score of 3, which indicates a higher level of systemic illness severity. In contrast, individuals who did not have any problems were distributed more evenly between ASA 2 and ASA 3 categories.*
	III	*30*	*18*	
	IV	*7*	*1*	

Table S2 - Postoperative Outcomes and Complications

Category	Metric	With Complications	Without Complications	Observations
Hospital Stay (days)	Mean	15.05	9.75	The t-test shows a **statistically significant difference** in hospital stay duration between the two groups (p = 0.0014), indicating that patients with complications tend to have significantly longer hospital stays
	Standard Deviation	8.15	2.44	
	Median	13	9	
Estimated Blood Loss (ml)	Mean	265.41	169.64	Statistical analyses, comprising a t-test (p = 0.0046) and a Mann-Whitney U test (p = 0.0086), demonstrate that this disparity is statistically significant. The results indicate a correlation between increased blood loss during surgery and a heightened risk of postoperative complications.
	Standard Deviation	140.62	124.23	
	Median	300	200	
Preoperative CRP (mg/L)	Mean	28.92	19.07	No significant changes in preoperative CRP levels were seen between complications and noncomplications. Patients without difficulties had an average preoperative CRP level of 19.1 ± 60.2 mg/L, while those with issues had 28.9 ± 43.7 mg/L. An independent t-test showed no change in preoperative CRP levels between groups (t = -0.74, p = 0.46). Levene's statistic = 0.54, p = 0.47, demonstrating variance homogeneity. The findings suggest that preoperative CRP levels may not predict surgical problems.
	Standard Deviation	43.68	60.18	
	Median	6.3	5.0	
CEA Levels (ng/mL)	Mean	19.70	348.52	This suggests that although there are a few individuals without problems who exhibit significantly elevated CEA levels, the general distribution of CEA levels does not exhibit a substantial difference between the two groups when evaluating the central tendency (median).
	Standard Deviation	70.13	1823.90	
	Median	3.19	2.52	
CA 19-9 Levels (U/mL)	Mean	57.81	235.91	Similar to the CEA analysis, the mean CA 19-9 level is much higher in patients without complications, largely due to some very high outlier
	Standard Deviation	185.54	1156.82	
	Median	10.30	12.66	
